# Automated AI fracture detection in initial presentation pediatric wrist X-rays: effects and benefits of adding follow-up examinations

**DOI:** 10.1007/s11547-025-02153-1

**Published:** 2025-11-13

**Authors:** Mario Scherkl, Nikolaus Stranger, Andreea Ciornei-Hoffman, Georg Singer, Tristan Till, Holger Till, Franko Hržić, Sebastian Tschauner

**Affiliations:** 1 Division of Pediatric Radiology, Department of Radiology, Medical University of Graz, Auenbruggerplatz 34, Graz, 8036 Styria Austria; 2 Department of Pediatric and Adolescent Surgery, Medical University of Graz, Auenbruggerplatz 34, Graz, 8036 Styria Austria; 3 University of Rijeka, Center for Artificial Intelligence and Cybersecurity, University of Rijeka, Radmile Matejcic 2, Rijeka, 51000 Primorsko-Goranska Croatia; 4 Department of Orthopaedic Surgery and Sports Medicine, Boston Children’s Hospital, Harvard Medical School, 300 Longwood Ave, Boston, 02445 MA USA

**Keywords:** Artificial intelligence, Fracture, Digital radiography, Aftercare, Trauma, Wrist, Pediatric radiology

## Abstract

**Background:**

Artificial Intelligence (AI) in radiology has shown promise in detecting fractures on initial X-rays. However, the role of follow-up examinations in enhancing AI performance remains unexplored. This study evaluates the impact of including follow-up X-rays on the performance of neural networks in detecting pediatric wrist fractures.

**Methods:**

Using the publicly available GRAZPEDWRI-DX dataset of 20,327 pediatric wrist X-rays, we created four training datasets: initial X-rays alone and combinations with follow-up X-rays (with and without casts). Two neural networks, EfficientNet (image classification) and YOLOv8 (object detection), were trained and evaluated using precision, recall, F1 score, and AP metrics. The dataset was divided into training, validation, and test sets, with 500 initial X-rays separated and reserved for testing.

**Results:**

EfficientNet models showed no statistically significant improvements in classification performance with the inclusion of follow-up X-rays. In contrast, YOLOv8 demonstrated improved object detection metrics, particularly AP50 (*p* = 0.003) and F1 score (*p* = 0.009), when follow-up X-rays were included. The improvement was most evident when both cast and non-cast follow-ups were incorporated.

**Conclusion:**

Adding follow-up X-rays did not enhance classification performance but improved fracture localization in object detection tasks. These findings suggest that including follow-up data shows no relevant improvement in the detection rate of fractures but can enhance AI applications for pediatric wrist fracture detection, particularly for object detection models.

## Introduction

Applications of artificial intelligence (AI) have achieved astonishing results in the medical field [1–3]. There are now plenty of algorithms in use in radiology that promise improvements in referral, image acquisition, image processing, post-processing, and reporting [4, 5]. Well-trained AI algorithms, particularly convolutional neural networks, have demonstrated remarkable capabilities in identifying anomalies in X-ray images with high precision, accuracy, and speed [6–13]. AI capabilities for fracture detection and localization remain an active area of research.

In terms of automated fracture detection or localization, this specifically means that initial X-rays from the first patient visit are evaluated [10, 13]. All further examinations after the injury has been diagnosed are not considered, despite the fact that they represent a substantial part of all available related X-rays. These follow-up examinations might regularly contain clinically relevant findings, such as complication during trauma healing such as secondary fracture dislocations, delayed or missing bone consolidation, metal implant problems, or infections [14, 15]. Despite the mentioned relevance, related AI applications are not very desirable to develop and promising to implement due to the comparably low incidence. Still, the topic will be of interest in the future and warrants further investigation.

Labeling image data is complex and time-consuming. Regularly, only experts can carry out these annotations, or at least need to supervise non-specialists or trainees [16–18]. It is, therefore, of great interest to gain an estimation which images actually need to be labeled so that the corresponding question can be answered more precisely by AI. Despite the great progress that has been made in the field of AI fracture detection [7, 19, 20], it is still unknown whether it makes sense to annotate an entire available dataset including follow-ups or not. No information on this can be found in the literature, but usually, practice by machine learning engineers is to use as much data as possible, ignoring potentially introduced biases. The main reason for this is that machine learning algorithms (especially deep convolutional neural networks) benefit from large datasets [21]. Longitudinal studies on the topic of AI and X-rays are relatively rare overall [22], not least due to the additional complexity of the tasks caused by the dimension of time.

To address the impact of adding follow-up examinations on machine learning models’ performance, the GRAZPEDWRI-DX dataset was utilized. The “GRAZPEDWRIDX” is a publicly available dataset specifically designed for machine learning applications in pediatric wrist trauma. This dataset consists of 20,327 wrist X-ray images from 6091 pediatric patients, including those with wrist fractures and other conditions such as osteopenia and soft tissue swelling. The dataset aims to support the development of machine learning models for automatic detection and diagnosis of wrist fractures in children, addressing the need for better diagnostic tools in pediatric radiology [23].

Our hypothesis was that there could be performance benefits in the initial presentation X-rays by adding annotated data of follow-up examinations into the AI training. We, therefore, tested the two standard neural networks for image classification and object detection with four different dataset variations containing either initial or combinations of initial and follow-up studies.

## Materials and methods

### Dataset

The utilized dataset GRAZPEDWRI-DX is freely available and composed of 20,327 pediatric wrist X-ray images [23]. It has been acquired between 2008 and 2018 at the University Hospital Graz, Austria, and contains digital 16-Bit pediatric wrist X-ray images from 6091 unique patients aged 0.2–19 years. The basic dataset included initial and follow-up X-ray images with and without cast. All X-ray images were reviewed by experienced pediatric radiologists, and diagnosed fractures were pre-labeled with bounding boxes, thus representing our ground truth data. From the basic dataset, 500 X-ray images were separated and served as the *test* subset that was not involved in any training process. The remaining 19,827 X-ray images could be grouped as follows:

- **Initial** (10,859 X-ray images at initial hospital presentation)
- **Follow-up no cast** (3692 follow-up X-ray images without a cast)
- **Follow-up cast** (5776 follow-up X-ray images with a cast)

**Fig. 1:**
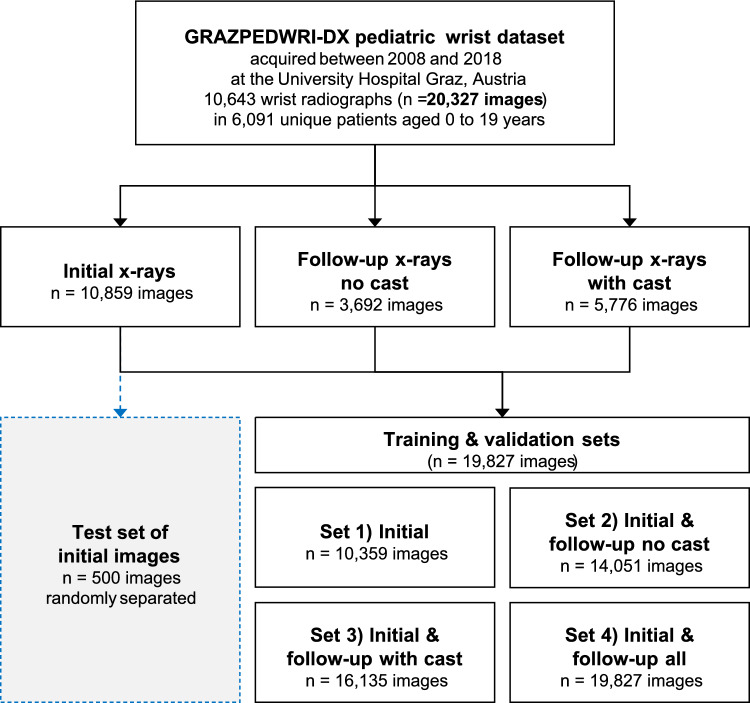
Flowchart of the study dataset and splits. Training datasets were combined from the three basic datasets after separating 500 initial images, resulting in four training datasets that underwent EfficientNet and YOLOv8 CNN training. We used the public dataset GRAZPEDWRI-DX for this systematic study [23]

The ratio between follow-up no cast to follow-up cast was 1–1.6 (39–61%). From our three basic datasets, we created *four different model datasets*, see Fig. 1, which we used to train our neural networks (YOLOv8 Nano to X-Large and EfficientNet B0–B7) and to test our hypothesis.

- **Initial** (10,359 X-ray images)
- **Initial + Follow-up no cast** (14,051 X-ray images)
- **Initial + Follow-up cast** (16,135 X-ray images)
- **Initial + Follow-up no cast + Follow-up cast** (19,827 X-ray images)

Before training, the training and validation subsets (without *n* = 500 images from the test set) were divided into two subsets: **train** (75% of the X-ray images in the model dataset) and **validation** (the remaining 25%). For EfficientNet, the provided parameter *valid pct* was set to 0.25 to perform a random split with 25% validation data. For YOLOv8, the X-ray images were randomly divided into the train (75%) and validation (25%) subfolders using an external Python script.

For the subset analysis, we divided the dataset in children and adolescents. Children were younger than 13 years, and adolescents were 13 years or older.

### Neural networks

**Fig. 2:**
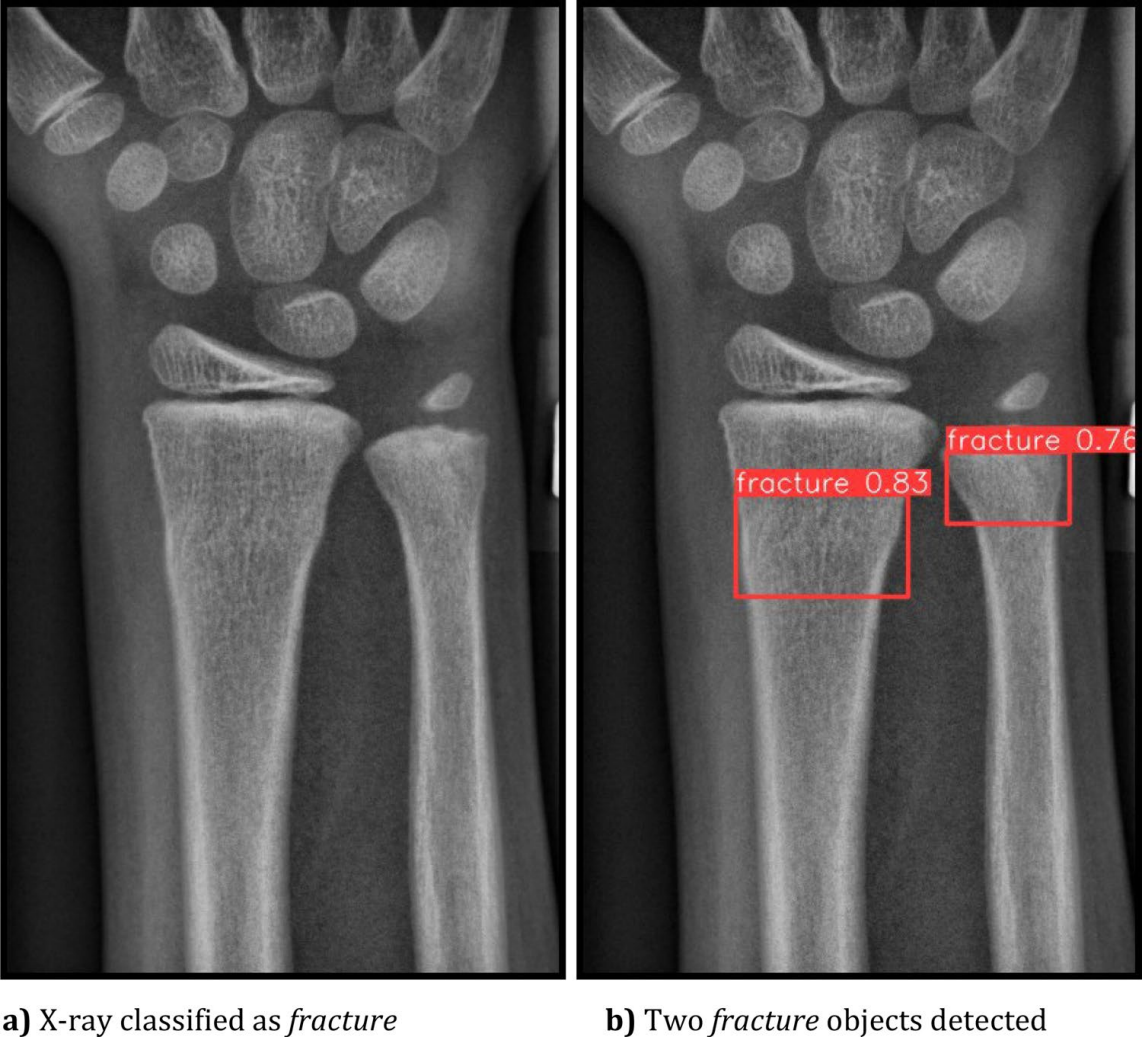
The different methods of classification, an object detection. Image from the ”GRAZPEDWRIDX” dataset [23]. EfficientNet image classification (without heatmap) on the left side and YOLOv8 object detection on the right side

Two neural network architectures were trained with the same ground truth data. Both represent different approaches to solving visual recognition tasks. The first architecture, **EfficientNet** [24], is designed for image classification tasks with a focus on efficiency in terms of both accuracy and computational resources, while the second, **YOLOv8** [25], focuses on object detection. For both visual recognition tasks, it is crucial to understand the visual content of images and extract meaningful features in order to make accurate predictions. Figure 2 illustrates the difference between object detection and image classification.

#### EfficientNet

EfficientNet comprises a series of convolutional neural network models designed for optimal utilization of computational resources in **image classification** tasks. For each X-ray image, the information as to whether there is a fracture or not is stored in a single CSV file. In order to train the model, the algorithm iterates over all X-ray images and links them with the associated information from the CSV file. Same as for YOLOv8, the test subset is used to evaluate the final performance of the trained model on unseen ground truth data. The entire training process is shown schematically in the supplementary material.

The EfficientNet family encompasses variants from EfficientNet-B0 to EfficientNetB7. The model’s index or letter in its name signifies the degree of scaling applied to the network architecture.

We used the **accuracy** metric to evaluate our EfficientNet image classification models. Accuracy refers to the effectiveness of a trained model in correctly identifying and assigning labels to X-ray images. In our work, all EfficientNet models were trained to deal with the binary classification problem *fracture* and consequently *no fracture*. A total of 32 different EfficientNet models were trained. The total number of training epochs was 50, and the target image size in pixel was set depending on the EfficientNet model to be trained: B0 = 224, B1 = 240, B2 = 260, B3 = 300, B4 = 380, B5 = 456, B6 = 528, and B7 = 600 px.

#### YOLOv8

YOLOv8 (You Only Look Once version 8) is an **object detection** architecture that operates by dividing an image into a grid and predicting bounding boxes and class probabilities for objects within each grid cell. The output is a set of bounding boxes that enclose the objects in the image, along with class labels and confidence scores for each box.

The model learns to detect fractures using all the pre-labeled X-ray images from the train subset. Each X-ray image is assigned an annotation file containing the coordinates of the bounding boxes for fractures. Throughout the training process, the performance of the model is regularly evaluated on the validation subset to guide the training process and prevent overfitting. Finally, the test subset is used to evaluate the final performance of the trained model on unseen ground truth data, providing an estimate of its generalization ability. The entire training process is shown schematically in the supplementary material.

Each category of YOLOv8 models comprises five distinct models. YOLOv8 Nano stands out as the swiftest and most compact option, while YOLOv8 X-Large claims the title of the most accurate but relatively slower model within the range.

We used the AP50 as our primary performance parameter because the focus of this study was to detect the class fracture but also used AP75 and AP50-95 metric to evaluate our YOLOv8 object detection models. It measures the mean average precision across IoU (Intersection over Union) thresholds from 0.5, 0.75 and 0.5 to 0.95, providing a comprehensive assessment of model performance. Every YOLOv8 model was trained from scratch to detect the class *fracture*. Fracture types or fracture locations were not further differentiated. A total of 20 YOLOv8 models were trained. The number of training epochs was 50, and the target image size was set to 640 px for each YOLOv8 model. Standard hyperparameters were used for the remaining parameters.

### Infrastructure

The training of both neural network architectures and their variants were performed at the authors institution on a Linux workstation equipped with two Nvidia GeForce RTX 4090 (video memory size of 24,209 MiB). A detailed system configuration is shown below.

- **OS:** Ubuntu 22.04 (Linux-6.5.0–14-generic- × 86 64-with-glibc2.35)
- **Framework:** Python-3.10.12 torch-2.0.1 + cu117
- **Neural networks:** Ultralytics YOLOv8.0.207, EfficientNet FastAI v2.7.15
- **GPU:** 2 × NVIDIA GeForce RTX 4090, 24.209MiB
- **CPU:** 16 core x 13th Gen Intel Core i7-13700 K
- **RAM:** 2 × 32 GB DDR4 RAM

### Statistical analysis

We performed a descriptive statistics and comparisons of means. Due to the potential missing normal distribution, we used non-parametric tests, specifically a Kruskal–Wallis tests to demonstrate differences between the dataset variants, using per variant performance values as unit of analysis. *P* values below 0.05 were assumed to be statistically significant. The Dunn's test and the Bonferroni correction for multiplicity correction were used for the post hoc tests. The corrected α using the Bonferroni correction method is 0.008333. SPSS version 27 (IBM Inc., Armonk, NY USA) was used to calculate the statistical parameters.

### Ethical statement

The retrospective data analysis was authorized by the ethics committee of the Medical University of Graz (IRB00002556, No. 31-108 ex 18/19). Due to the retrospective nature of the study, informed consent was not required. All experimental procedures complied with both local guidelines and the Declaration of Helsinki.

## Results

### Classification metrics (EfficientNet)

**Fig. 3:**
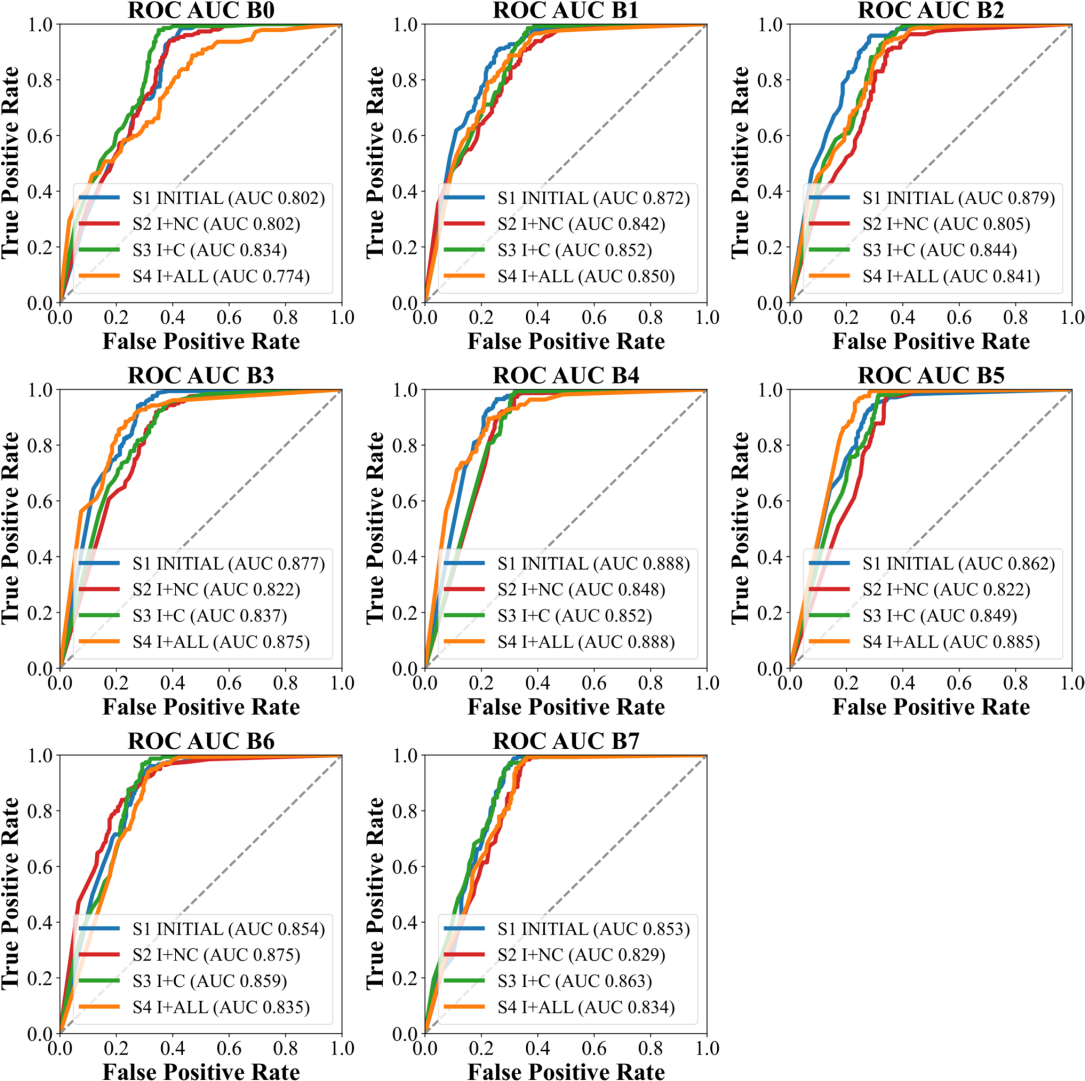
ROC analyses of the four training variants in the test set of 500 initial images

The test results obtained for our primary endpoint, the ROC analyses are shown in Fig. 3, showing no visual trends or difference between the four training datasets. We did not detect statistically significant differences between the four training datasets in the test set composed of 500 initial images by non-parametric Kruskal–Wallis tests across the eight EfficientNet variants, Precision (*p* = 0.252), Recall (*p* = 0.185), Accuracy (*p* = 0.088), and F1 score (*p* = 0.136). Therefore, further post hoc tests were not performed. This means that there was no identifiable benefit from the inclusion of additional follow-up X-rays with respect to the classification between the presence and exclusion of a fracture. We further divided the dataset in children and adolescents were the children subset showed slightly better performance parameters, shown in Table 1.

**Table 1:** The table shows the performance parameters of the subset analysis with slightly better performance parameters in the children subset

Model	Children	Adolescents	Combined	Children	Adolescents	Combined	Children	Adolescents	Combined
	Precision			Recall			F1		
initial_follow-up_cast_follow-up_no cast_l	0.935	0.950	0.950	0.907	0.819	0.866	0.921	0.867	0.906
initial_follow-up_cast_follow-up_no cast_m	0.966	0.942	0.957	0.886	0.781	0.859	0.924	0.847	0.905
initial_follow-up_cast_follow-up_no cast_n	0.967	0.928	0.958	0.865	0.781	0.844	0.913	0.842	0.897
initial_follow-up_cast_follow-up_no cast_s	0.946	0.927	0.945	0.893	0.763	0.853	0.919	0.834	0.897
initial_follow-up_cast_follow-up_no cast_x	0.964	0.925	0.950	0.906	0.831	0.897	0.934	0.880	0.923
initial_follow-up_cast_l	0.969	0.956	0.962	0.882	0.782	0.861	0.923	0.847	0.909
initial_follow-up_cast_m	0.972	0.956	0.965	0.873	0.790	0.850	0.920	0.850	0.904
initial_follow-up_cast_n	0.954	0.844	0.941	0.890	0.783	0.849	0.921	0.846	0.893
initial_follow-up_cast_s	0.927	0.892	0.924	0.911	0.795	0.878	0.919	0.852	0.900
initial_follow-up_cast_x	0.967	0.943	0.965	0.873	0.792	0.850	0.918	0.850	0.904
initial_follow-up_no cast_l	0.956	0.928	0.939	0.890	0.771	0.861	0.922	0.840	0.898
initial_follow-up_no cast_m	0.942	0.953	0.930	0.890	0.771	0.866	0.915	0.837	0.897
initial_follow-up_no cast_n	0.957	0.929	0.953	0.844	0.791	0.832	0.897	0.841	0.888
initial_follow-up_no cast_s	0.970	0.914	0.954	0.869	0.772	0.841	0.917	0.838	0.894
initial_follow-up_no cast_x	0.946	0.942	0.945	0.895	0.783	0.866	0.920	0.846	0.904
initial_l	0.941	0.859	0.922	0.873	0.807	0.853	0.906	0.854	0.886
initial_m	0.954	0.921	0.941	0.878	0.771	0.850	0.914	0.837	0.893
initial_n	0.938	0.915	0.926	0.890	0.777	0.861	0.913	0.840	0.892
initial_s	0.917	0.925	0.937	0.886	0.783	0.836	0.901	0.838	0.884
initial_x	0.945	0.903	0.908	0.876	0.783	0.866	0.909	0.841	0.887

Model	Children	Adolescents	Combined	Children	Adolescents	Combined	Children	Adolescents	Combined
	mAP50			mAP75			mAP50-95		
initial_follow-up_cast_follow-up_no cast_l	0.944	0.890	0.930	0.717	0.682	0.699	0.625	0.581	0.612
initial_follow-up_cast_follow-up_no cast_m	0.946	0.887	0.931	0.689	0.703	0.687	0.623	0.579	0.610
initial_follow-up_cast_follow-up_no cast_n	0.935	0.880	0.921	0.710	0.561	0.681	0.610	0.544	0.594
initial_follow-up_cast_follow-up_no cast_s	0.941	0.874	0.927	0.700	0.607	0.673	0.626	0.552	0.607
initial_follow-up_cast_follow-up_no cast_x	0.952	0.905	0.940	0.743	0.652	0.718	0.638	0.577	0.621
initial_follow-up_cast_l	0.942	0.873	0.924	0.714	0.694	0.704	0.621	0.556	0.603
initial_follow-up_cast_m	0.942	0.868	0.923	0.694	0.571	0.666	0.614	0.539	0.594
initial_follow-up_cast_n	0.938	0.860	0.918	0.690	0.603	0.668	0.607	0.542	0.589
initial_follow-up_cast_s	0.948	0.871	0.929	0.726	0.619	0.700	0.623	0.553	0.605
initial_follow-up_cast_x	0.940	0.875	0.923	0.689	0.620	0.672	0.625	0.541	0.604
initial_follow-up_no cast_l	0.936	0.865	0.919	0.703	0.616	0.680	0.618	0.531	0.596
initial_follow-up_no cast_m	0.935	0.879	0.921	0.683	0.603	0.660	0.616	0.563	0.600
initial_follow-up_no cast_n	0.921	0.873	0.909	0.712	0.667	0.695	0.607	0.549	0.591
initial_follow-up_no cast_s	0.933	0.878	0.920	0.688	0.616	0.670	0.608	0.562	0.595
initial_follow-up_no cast_x	0.934	0.874	0.919	0.693	0.595	0.667	0.620	0.546	0.600
initial_l	0.934	0.865	0.917	0.690	0.590	0.663	0.611	0.549	0.594
initial_m	0.933	0.852	0.913	0.678	0.578	0.650	0.604	0.518	0.582
initial_n	0.935	0.856	0.916	0.692	0.614	0.679	0.617	0.550	0.600
initial_s	0.930	0.852	0.911	0.686	0.553	0.653	0.607	0.518	0.585
initial_x	0.933	0.869	0.917	0.710	0.594	0.682	0.617	0.544	0.600

### Object detection metrics (YOLOv8)

**Fig. 4:**
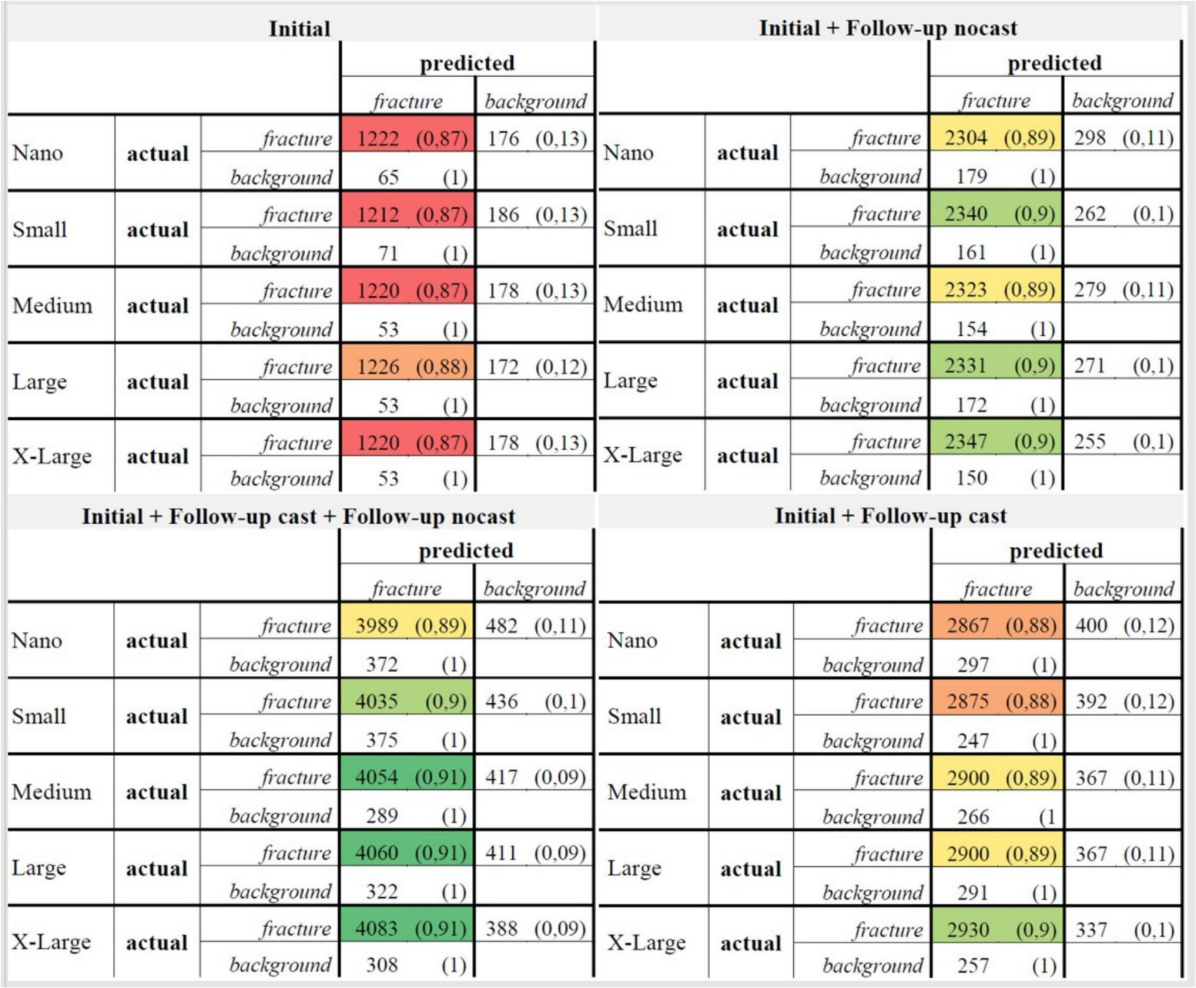
Confusion matrices of the Initial dataset, Initial + Follow-up no cast, Initial + Follow-up cast + Follow-up no cast, and Initial + Follow-up cast, grouped by model

**Fig. 5:**
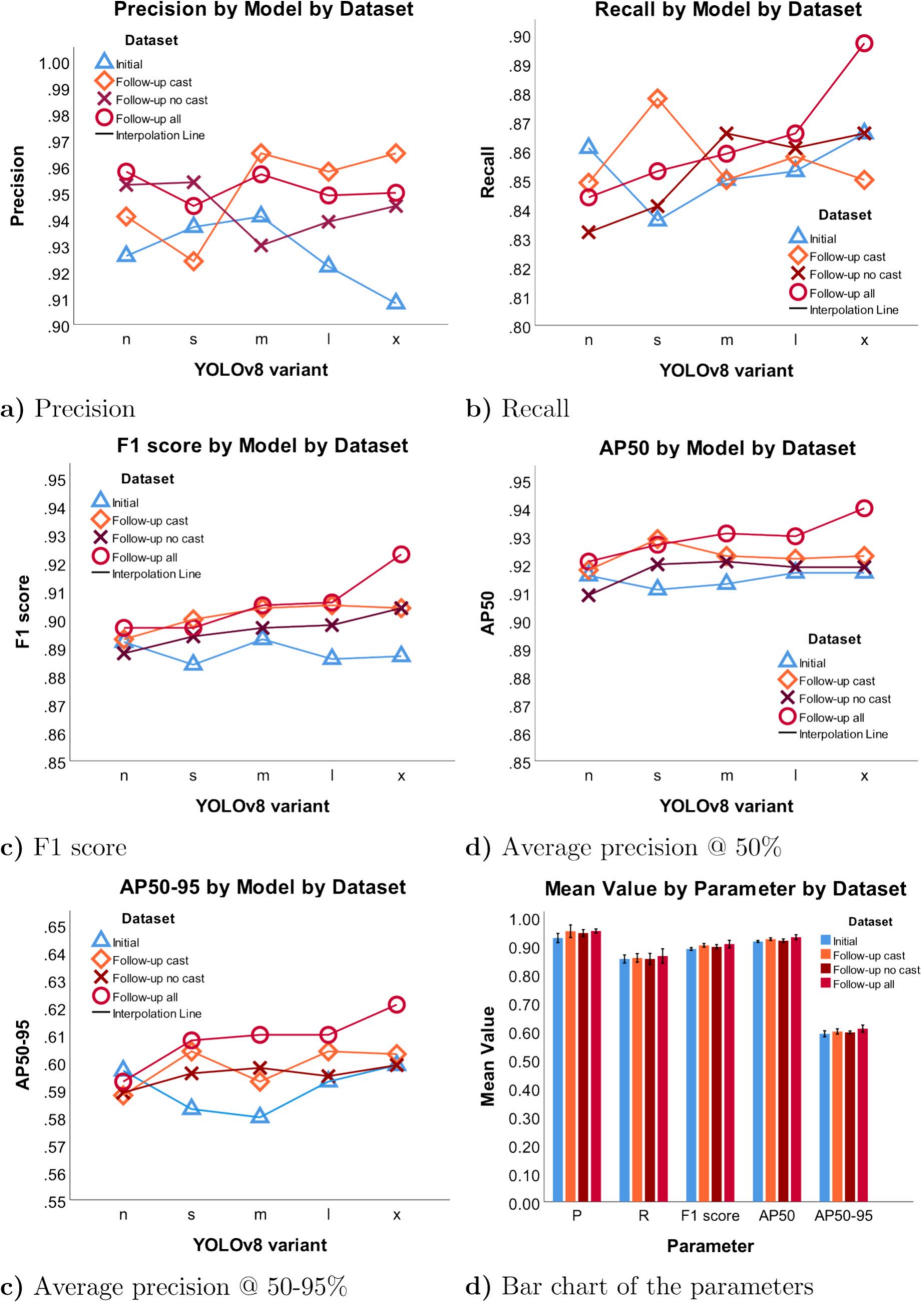
Scatter plots of YOLOv8 variant metrics in the test set of 500 initial images. About 10% ranges of values are given for the scatter plots. The bar chart displays the full range of values, in order to put the differences into perspective

The object detection algorithm YOLOv8 with its five variants *n, s, m, l*, and *x* demonstrated statistically significant differences between the training datasets in independent-samples Kruskal–Wallis test with post hoc Dunn's test using a Bonferroni corrected alpha of 0.0083, namely, regarding AP50 (*p* = 0.003), F1 score (*p* = 0.009), and Precision (*p* = 0.038). AP50-95, AP75, and Recall were not statistically different between the training datasets with *p* = 0.064, *p* = 0.122, and *p* = 0.871, Fig. 5. The associated confusion matrixes are shown in Fig. 4.

## Discussion

We investigated the possible effects of additional follow-up examinations on the performance metrics for AI-assisted fracture detection on pediatric wrist X-rays at the initial visit. Numerous follow-up examinations are available in many hospitals, but their use for AI solutions requires expert image labeling, which is time-consuming and labor-intensive. This manuscript analyzed the effects of including follow-up radiographs, with and without a cast, in AI training. We aimed to determine whether the increased annotation efforts and the resulting larger database could affect AI detection performance on initial fracture radiographs, which is currently the only common use case in pediatric traumatology.

An extensive review of the literature was conducted, yet no studies were identified that addressed the use of follow-up imaging in the context of pediatric fracture recognition using AI. Information on the longitudinal aspects of pediatric trauma related to artificial intelligence is generally scarce. The incorporation of an additional temporal dimension presents challenges, and as a result, longitudinal studies are infrequently encountered within the domain of computer vision. However, some recent research has focused on follow-up applications involving chest radiographs [22, 26].

**Fig. 6:**
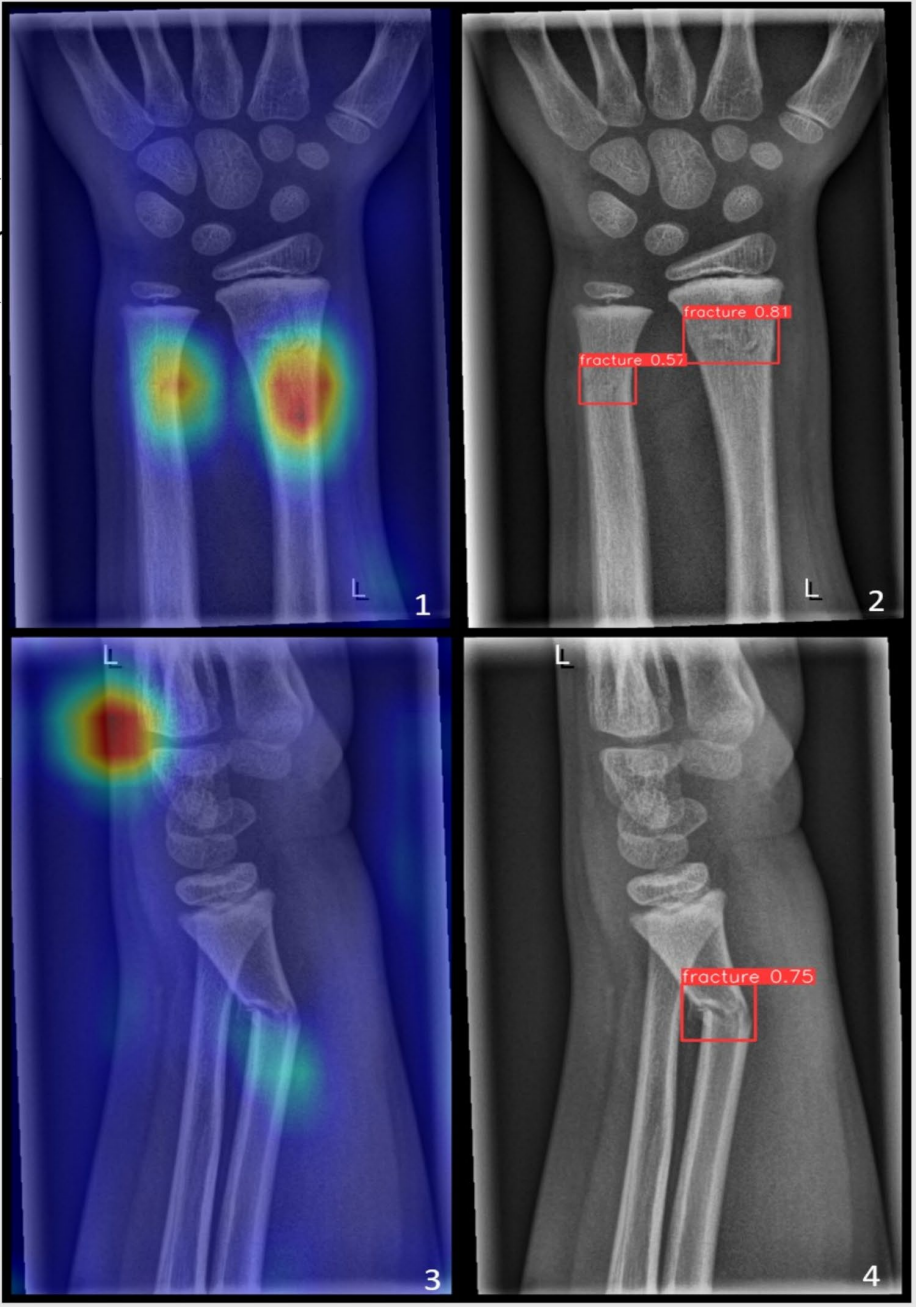
Figures 1 and 2 show that the existing fractures were predicted by both the EfficientNet image classification model in the first picture and the YOLOv8 object detection model in the second picture. The EfficientNet image classification model in picture 3 has incorrectly classified the X-ray of an obvious fracture as not being a *fracture*. Compared to a correctly detected fracture by the YOLOv8 object detection model in picture 4. Image from the ”GRAZPEDWRI-DX” dataset [23]

Current AI applications and research in the field if automated X-ray-based fracture detection focuses only on the initial patient visit [10, 13], despite the clinical relevance of follow-up imaging. Therefore, the whole spectrum of follow-up examinations is left aside. It is not uncommon that these follow-up studies are more important for patient outcomes than the first images. This is the case when repositioning might be insufficient, in case of secondary dislocation, instrumentation complications arise, or if post-traumatic infections occur. There is also little research on pathological fractures, as their occurrence is low, impairing the current deep learning algorithms requirement for large numbers of samples. Examples of correctly classified and detected fractures in the test set are given in Fig. 6.

We trained all eight variants of EfficientNet as image classification algorithm on the freely available GRAZPEDWRI-DX dataset [23] which had been published by our group before. While we observed a trend of increased performance metrics with increasing model complexity, we did hardly notice any relevant differences between our four training and validation set variants. This indicates that the inclusion of follow-up images did not add any value with regard to the detection of pediatric fractures. Conversely, this would also mean that the effort of image labeling can be omitted in the context of pediatric wrist fracture detection by classification.

Interestingly, the YOLOv8 variants showed improved fracture localization when follow-up images came into consideration. This was also statistically significant between two datasets in post hoc testing of the non-parametric Kruskal–Wallis analysis over all five variants, specifically between 1) Initial and 4) Follow-up all (*p* = 0.003) for the AP50 metric, and between 1) Initial and 2) Follow-up cast (*p* = 0.047) and 1) Initial and 4) Follow-up all (p = 0.009) for the F1 score metric. Other metrics trended toward statistical significance. The least observable difference in YOLOv8 was between the test sets 1) Initial and 3) Follow-up no cast. In contrast with fracture classification, fracture detection with YOLOv8 improved on the test set of initial X-rays by adding more diverse data in terms of follow-up samples. A test set example, where classification failed but object detection functioned correctly, is demonstrated in Fig. 6.

Several limitations of this study merit recognition. Firstly, the dataset is derived exclusively from a single institution which prohibits further analyses in regard to generalization of the findings. Secondly, the data pertain solely to one body region. It should also be noted that there is significant skewness in the distribution of image groups within the dataset, as the numbers of initial follow-up and cast images were not evenly distributed. This distribution was caused by a lack of samples. Furthermore, the casts in the follow-up X-rays could skew the results by decreasing the accuracy of setting the bounding boxes in the ground truth. A further limitation is also the lack of external validation. Further specific research will be necessary to elucidate the characteristics of initial studies and follow-up examinations in the domain of AI and pediatric trauma radiography.

## Conclusions

Automatic fracture detection as an increasingly important AI application has so far been based on initial X-ray images almost exclusively. The follow-up X-rays did not improve classification performance on the initial X-rays but positioning performance of the boxes improved in object detection tasks. However, the results of the study show that there is no relevant improvement in the detection rate of childhood wrist fractures by including additional follow-up X-rays, but could show improved object detection. Therefore, additional effort in labeling follow-up examinations should be carefully evaluated about the intended task and annotation resources.
